# Prevalence and Risk Factors of Mycoplasma Hyopneumoniae in Swine Farms, Mainland China, 2003–2024: A Meta-Analysis

**DOI:** 10.3390/vetsci12090863

**Published:** 2025-09-05

**Authors:** Hongyu Zhou, Huiling Zhang, Xueping Zhang, Lina Ye, Xinyuan Liu, Tangjie Zhang

**Affiliations:** 1 College of Veterinary Medicine (Institute of Comparative Medicine), Yangzhou University, Yangzhou 225009, China; zhou2096177403@foxmail.com (H.Z.); zhangxp1995@foxmail.com (X.Z.); 201901123@stu.yzu.edu.cn (L.Y.); 18795961172@163.com (X.L.); 2 Jiangsu Co-Innovation Center for Prevention and Control of Important Animal Infectious Diseases and Zoonoses, Yangzhou 225009, China; 3 Independent Researcher, New York, NY 11355, USA; huz21@pitt.edu

**Keywords:** *Mycoplasma hyopneumoniae*, swine, prevalence, meta-analysis, mainland China, epidemiology

## Abstract

This is the first systematic review and meta-analysis on the prevalence and risk factors of *M. hyopneumoniae* infection in swine farms across mainland China from 2003 to 2024. A total of 54 eligible cross-sectional studies were analyzed by stratifying farms as subclinically or clinically infected. The overall pooled prevalence of *M. hyopneumoniae* was estimated as 33.4%, with clinical infection farms showing a significantly higher prevalence (52.9%) than subclinical farms (11.5%). Subgroup analyses revealed significant variations in infection rates based on age, sampling year, geographic region, farming scale, season, sampling type, and diagnostic method. Small-scale farms, farms with breeding swine, and farms in the Northwest region showed the highest infection rates. Diagnostic methods and sampling types also significantly influenced detection rates. Sensitivity analyses confirmed the robustness of the results, while publication bias was addressed using the Trim-and-Fill method. To effectively reduce the burden of *M. hyopneumoniae* in the swine industry in mainland China, future efforts should prioritize enhanced biosecurity, improved diagnostic accuracy, and region-specific vaccination and management strategies.

## Simple Summary

*Mycoplasma hyopneumoniae* (*M. hyopneumoniae*) is a major respiratory pathogen in pigs, causing significant economic losses to swine production. This study systematically evaluated its prevalence and risk factors in swine farms across mainland China from 2003 to 2024. The pooled prevalence was 33.4%, with higher rates in clinically affected farms (52.9%) than subclinical ones (11.5%). Infection rates varied by region, age, farm scale, diagnostic method, and sample type. This research highlights the need for region-specific biosecurity, vaccination strategies, and diagnostic improvements to better control the disease and reduce its impact on the swine industry.

## 1. Introduction

*M. hyopneumoniae* is a key respiratory pathogen in swine and the causative agent of a common, widely distributed disease that occurs year-round. It is restricted to swine and is present in all major swine-raising countries, causing tremendous global financial losses to the swine industry due to its role in porcine respiratory disease complex (PRDC) [1,2]. The primary losses stem from the costs associated with treatment and vaccination, as well as reduced performance and higher mortality rates resulting from secondary infections [3].

*M. hyopneumoniae*, a species within the genus *Mycoplasma*, lacks a cell wall, which enhances its tolerance to external environmental conditions and enables its survival under various conditions, particularly in dry and warm environments. Additionally, it exhibits intrinsic resistance to many commonly used antibiotics, such as penicillin [4,5]. *M. hyopneumoniae* is primarily transmitted via airborne routes, with an increased risk of spread in high-density farming environments. Prolonged exposure to environmental stressors, including overcrowding and poor ventilation, can exacerbate disease severity. Additionally, direct contact between swine serves as a significant mode of transmission [6]. As *M. hyopneumoniae* infection progresses, it stimulates a sustained immune response that contributes to chronic respiratory disease, characterized by persistent inflammation of the airways. In parallel, the pathogen can interfere with innate defense mechanisms such as mucociliary clearance, which facilitates long-term colonization. This dual effect leads to a significant decline in production performance and immune function, accompanied by clinical symptoms such as coughing, tachypnea, and elevated body temperature [7]. Moreover, the chronic and recurrent nature of Mycoplasma infections presents significant challenges to disease prevention and control in swine farms and has led to substantial economic losses in the global swine industry [8,9,10]. *M. hyopneumoniae* infection is highly prevalent in domestic swine populations and has been reported in nearly all swine-producing regions, with infection rates ranging from 38% to 100% [11].

A peculiar feature of pig farming in mainland China is the coexistence of modern intensive production systems with a large number of smallholder and backyard farms. Currently, Mycoplasmal Pneumonia of Swine (MPS) is widely prevalent in swine farms across mainland China, typically occurring in a sporadic manner. In certain regions with suboptimal farming conditions and unfavorable climates, MPS may exhibit an endemic trend [12,13]. Due to characteristics such as the prolonged infection period, variable incubation time, potential for long-distance transmission, and antimicrobial resistance, complete eradication of the disease from swine farms remains challenging. The current control measures for *M. hyopneumoniae* infections in swine include vaccination and antibiotic treatment. Vaccination is a key strategy in managing the disease; however, its effectiveness can vary due to factors such as the alignment between vaccine strains and field strains, as well as the immunization protocols used [14]. Although antibiotic treatment can help control the infection to some extent, it also raises concerns about antimicrobial resistance [15,16]. Additional control measures involve optimizing management practices and strengthening biosecurity [1,8].

Given the significant differences in the clinical manifestations and epidemiological characteristics of *M. hyopneumoniae* infection in swine farms across mainland China, this study categorizes farms into two groups: subclinical infection farms and clinical infection farms. Clinical infection farms refer to herds in which *M*. *hyopneumoniae* infection is present but without evident clinical respiratory signs; infection is usually detected through laboratory tests or by characteristic lung lesions at slaughter. By contrast, subclinical infection farms are those in which pigs show no clinical signs and no evidence of infection was detected during sampling. This stratification aims to more accurately capture variations in prevalence estimates across different farm types.

The objectives of this study are to determine the prevalence of *M. hyopneumoniae* across different regions and farm types in mainland China, identify associated risk factors, and highlight high-risk areas to inform targeted prevention and control measures. Additionally, the study aims to provide evidence-based guidance for optimizing farm management and biosecurity practices, considering farm scale, pig age, and regional context, thereby reducing infection risk and supporting effective, differentiated control strategies.

## 2. Materials and Methods

### 2.1. Search Strategy

This study adhered to the PRISMA (Preferred Reporting Items for Systematic Reviews and Meta-Analyses) guidelines [17]. To ensure the inclusion of all relevant information in the analysis, the PRISMA checklist was applied (Supplementary Data S1).

All the literature conducted on the prevalence of *M. hyopneumoniae* in swine from January 2003 to January 2025 was searched using four databases, including English databases (PubMed and Web of Science) and Chinese databases (CNKI and Wan Fang databases). The Chinese search terms were “Mycoplasma pneumonia” and “Pig”. The English search terms were “Mycoplasma pneumonia”, “Pig” or “Swine”, and “China”. Additionally, the references of the published papers were checked to retrieve papers that were not found via database searching.

### 2.2. Inclusion and Exclusion Criteria

The full-text articles were retrieved, and studies were included in the review based on the following criteria: 1. Study location: Mainland China. 2. Study subjects: Swine. 3. Study design: Cross-sectional studies. 4. Detection methods: Clearly stated with a detailed description of procedures. 5. Sample size: Greater than 30 with accurate data. 6. Sample background: Information must be fully described and documented. 7. Publication status: No duplicate publications.

The literature review was conducted independently by two reviewers. In cases where the results were inconsistent, discrepancies were resolved either by consulting a third reviewer or through discussion and negotiation. Articles that did not meet the inclusion criteria were excluded.

The screened literature for *M. hyopneumoniae* prevalence was carefully read, and the following information was collected: the first author, year of publication, sampling year, geographic region, age, diagnostic method, sampling type, farming scale, total sample size, and number of positive samples. Although studies were identified from most provinces, certain regions such as Tibet, Ningxia, and Hainan were scarcely represented, reflecting the limited availability of published data.

### 2.3. Quality Assessment and Data Extraction

After removing duplicate records and review articles, the titles and abstracts of all remaining studies were screened to preliminarily exclude those unrelated to the prevalence of *M. hyopneumoniae* in swine. The full texts were then reviewed to determine eligibility based on the following criteria: (1) the study was conducted in mainland China; (2) the study population consisted of swine; (3) the article reported a cross-sectional study; (4) the diagnostic method and procedures were clearly described; (5) the number of clinical samples was greater than 10, and accurate data were available; (6) the background information of the samples was complete. Literature screening was independently performed by two reviewers. Studies not meeting the inclusion criteria were excluded. Disagreements or uncertainties regarding eligibility were resolved by a third reviewer or through discussion.

Cross-sectional studies in animals differ from randomized clinical trials. Their systematic evaluation methods are not yet fully established, and these studies typically lack control groups. Therefore, the systematic evaluation methods for animal cross-sectional studies were adapted from the Cochrane quality assessment framework. The quality of the studies was assessed by evaluating the following six criteria using RevMan 5.4. A score of 2 was assigned if a study met a given criterion, 1 if the criterion was unclear or not explicitly described, and 0 if it was not met. The quality assessment criteria included the following: 1. whether all relevant details were reported; 2. whether the study provided a detailed description of experimental procedures; 3. whether the sampling process was conducted randomly; 4. whether the study spanned multiple time points; 5. whether statistical analyses were performed; 6. whether missing data were reported and addressed; 7. whether the study evaluated potential risk factors. Each criterion was assigned equal weight in the overall quality assessment.

Studies with a total quality evaluation score below 7 points were excluded from the statistical analysis.

### 2.4. Statistical Analysis

Data processing was performed using Stata software (version 16), and the double-arcsine transformation (PFT) was applied to normalize the data distribution [18,19]. The heterogeneity of *M. hyopneumoniae* infection rates across the included studies was assessed using the I^2^ and Q tests. If *p* ≥ 0.10 and I^2^ < 50%, it suggests low or no statistical heterogeneity among the study effect sizes, and a fixed-effects model should be applied for the analysis. Conversely, if *p* < 0.10 and I^2^ ≥ 50%, it indicates significant statistical heterogeneity, warranting the use of a random-effects model to combine the estimated infection rates and their 95% confidence intervals (CIs) [20]. To determine whether publication bias exists in this study, a funnel plot was drawn, and publication bias is assessed using the Begg test or Egger test. If publication bias is detected, the Trim-and-Fill method can be applied for correction, followed by recalculating the overall effect size [21,22]. Furthermore, sensitivity analysis is performed to verify its robustness.

Subgroup analyses were performed to explore the sources of heterogeneity, followed by meta-regression analyses to investigate the risk factors for *M. hyopneumoniae* infection. The subgroups considered in the analyses included year of sample collection, sampling season, diagnostic methods, age group of swine, farming scale (large-scale, medium-scale, and small-scale), and farm type (subclinical infection farms and clinical infection farms).

## 3. Results

### 3.1. Literature Search Results

**Figure 1:**
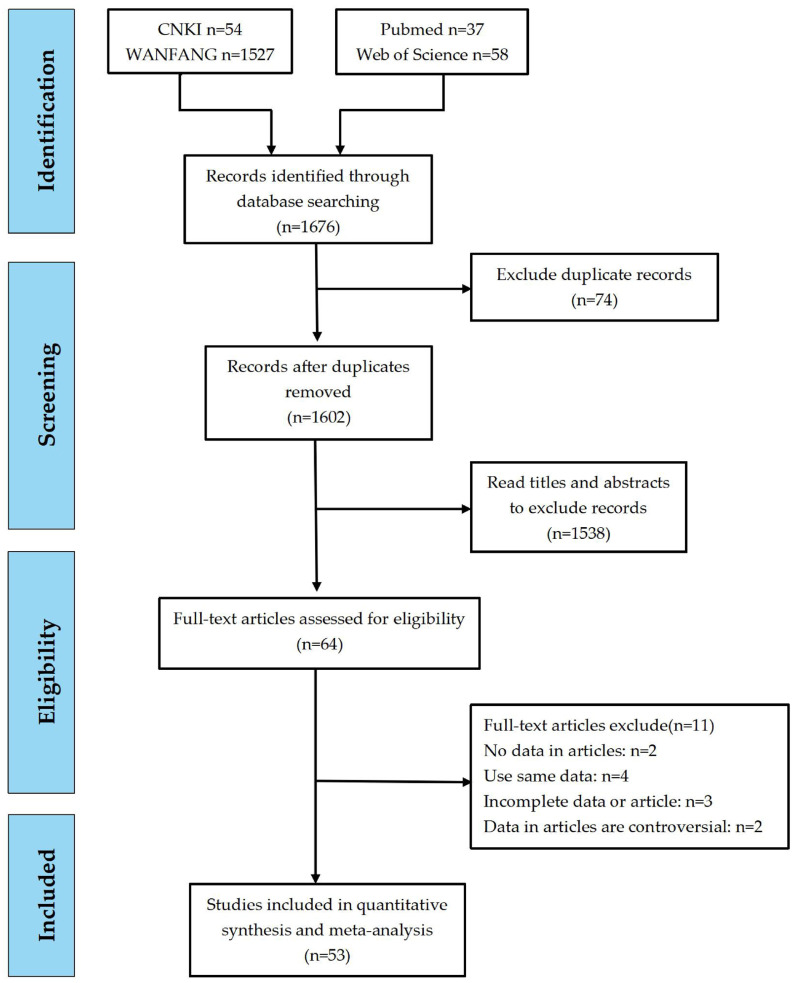
Flow diagram of the selection of eligible studies.

A total of 1676 articles were retrieved from four Chinese and English databases based on the keywords, including 54 from the China National Knowledge Infrastructure (CNKI), 1527 from Wanfang Data, 58 from Web of Science, and 37 from PubMed. After eliminating duplicate studies and following the screening process outlined in the Cochrane Handbook, articles were assessed based on their titles, abstracts, full texts, and predefined exclusion criteria. Ultimately, 54 articles were included for data analysis. The screening process and results are presented in Figure 1.

The data screened from the 54 studies on *M. hyopneumoniae* prevalence are shown in Table 1.

### 3.2. Data Extraction and Quality Analysis

The quality of each study was assessed based on a checklist of five items, and the corresponding scores are illustrated in Supplementary Data S2. Among the 54 included studies, 42 were rated as high quality, 12 as medium quality, and none as low quality.

### 3.3. Heterogeneity Analysis

**Figure 2:**
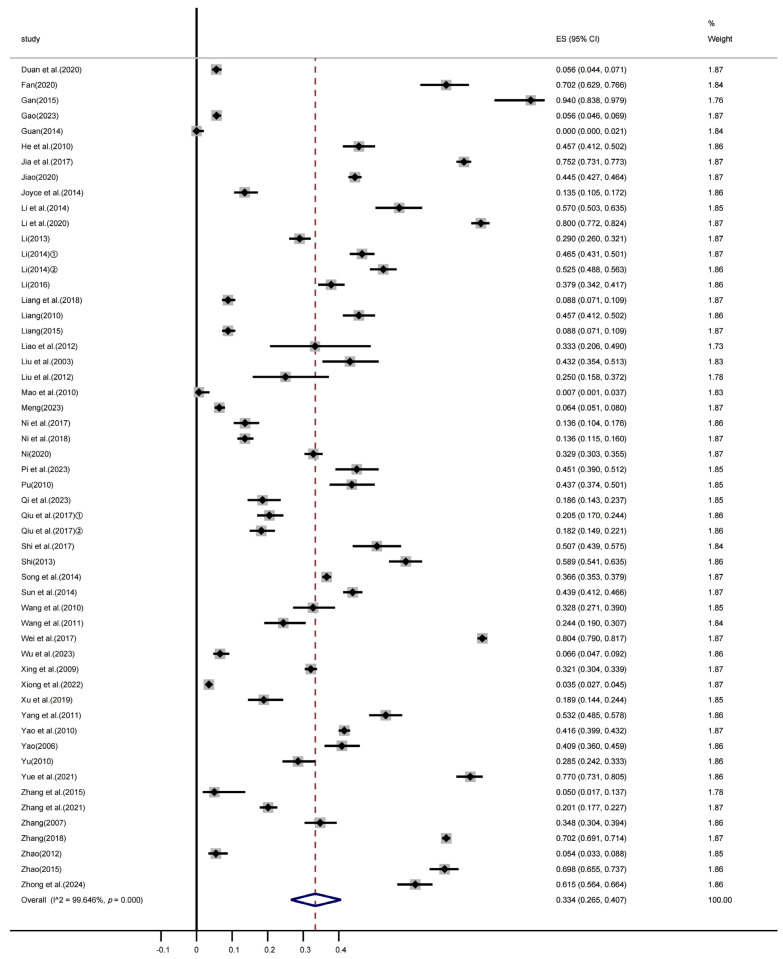
Summary forest plot of the estimated *M. hyopneumoniae* prevalence with random-effects analyses.

A meta-analysis was conducted based on data extracted from the final 54 included studies, revealing significant heterogeneity among the studies (χ^2^ = 14984.484, *p* < 0.001; I^2^ = 99.646%). A random-effects model was used to calculate the pooled prevalence, resulting in an estimated prevalence of 33.4% (95% CI: 26.5–40.7%). Figure 2 presents the forest plot of *M. hyopneumoniae* prevalence in China from 2002 to 2024.

The points represent the prevalence rates, and the horizontal lines represent the 95% confidence intervals corresponding to the effect sizes (ESs). The diamond shape at the bottom indicates the pooled effect size.

**Figure 3:**
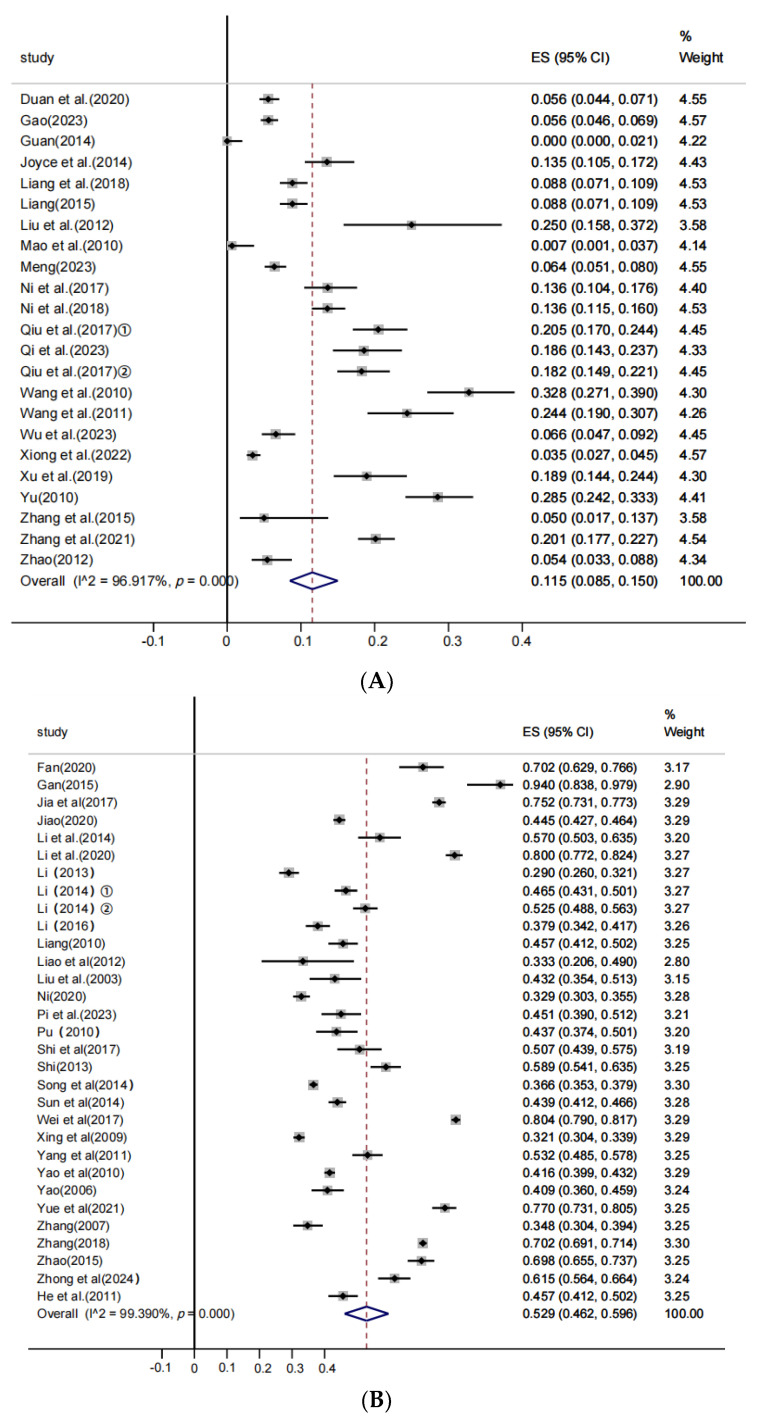
Forest plot of the prevalence of *M. hyopneumoniae* in swine with random-effects analysis in subclinical infection swine farms (**A**) and clinical infection swine farms (**B**).

The included studies were further stratified into subclinical infection farms and clinical infection farms. Based on 23 studies of subclinical infection farms, significant heterogeneity was observed (χ^2^ = 713.517, *p* < 0.001; I^2^ = 96.917%), and the estimated pooled prevalence calculated by the random-effects model was 11.5% (95% CI: 8.5–15.0%). Figure 3A shows the forest plot for *M. hyopneumoniae* prevalence in subclinical infection farms. Data from 31 studies on clinical infection farms also demonstrated significant heterogeneity (χ^2^ = 4917.036, *p* < 0.001; I^2^ = 99.390%). The pooled prevalence estimated using the random-effects model was 52.9% (95% CI: 46.2–59.6%). Figure 3B depicts the forest plot of *M. hyopneumoniae* prevalence in clinical infection farms.

### 3.4. Subgroup Analysis

Subgroup analysis was conducted to explore potential sources of heterogeneity, with results stratified by age, sampling year, geographic region, farming scale, season, sampling type, and diagnostic method, as presented in Table 2.

The possible causes of *M. hyopneumoniae* infection heterogeneity were analyzed. The studies were divided into subgroups on the basis of study age, sampling year, geographic region, farming scale, season, sampling type, and diagnostic method.

In clinically infected swine farms, significant differences in *M. hyopneumoniae* prevalence were observed among different age groups (*p* = 0.005). The infection rates, from highest to lowest, were as follows: breeding swine (61.7%), fattening swine (49.1%), suckling swine (38.0%), nursery swine (36.1%), and growing swine (32.7%). In contrast, no significant age-related differences were found in subclinically infected farms (*p* = 0.606), with prevalence rates of 13.7% in suckling swine, 20.7% in nursery swine, 11.7% in growing swine, 16.9% in fattening swine, and 17.2% in breeding swine. The overall pooled prevalence was estimated at 39.80% (95% CI: 34.1–45.6%).

In the subgroup analysis by sampling year, the prevalence of *M. hyopneumoniae* in clinically infected swine farms showed an increasing trend over time, with infection rates of 49.7% before 2013, 54.6% during 2014–2018, and 56.2% during 2019–2024. However, these differences were not statistically significant. In subclinically infected swine farms, the prevalence remained relatively stable across the three periods, ranging from 10% to 12%, with no significant differences observed.

In the regional subgroup analysis, significant differences in *M. hyopneumoniae* prevalence were observed among clinically infected swine farms (*p* = 0.005). The highest prevalence was reported in the Northwest region (64.2%), followed by the Qinghai–Tibet region (58.9%), the Northern region (55.7%), and the Southern region (47.4%). In subclinically infected swine farms, no significant regional differences were found (*p* = 0.272), with prevalence rates of 9.4% in the Northern region, 9.6% in the Southern region, and 11.8% in the Qinghai–Tibet region. Due to the availability of only one study from the Northwest region, it was excluded from the subclinical analysis. Overall, the prevalence in subclinically infected farms was significantly lower than that in clinically infected farms across all regions.

In the subgroup analysis based on farming scale, significant differences in *M. hyopneumoniae* prevalence were observed among farms of different scales (*p* = 0.012). The highest infection rate was observed in small-scale farms (≤100 swine) at 35.7%, followed by medium-scale farms (100–500 swine) at 27.9%, while large-scale farms (>500 swine) had the lowest prevalence at 2.3%.

Seasonal subgroup analysis showed no statistically significant differences in *M. hyopneumoniae* prevalence across seasons in either subclinically infected farms (*p* = 0.127) or clinically infected farms (*p* = 0.105). In subclinically infected farms, the prevalence was 16.6% in spring, 4.4% in summer, 5.0% in autumn, and 9.4% in winter. In clinically infected farms, the corresponding rates were 54.7% in spring, 36.0% in summer, 65.1% in autumn, and 47.1% in winter.

The subgroup analysis based on sampling type revealed significant differences in clinically infected swine farms (*p* = 0.033). The highest prevalence was observed in tissue samples (56.8%), followed by serum samples (44.6%), with the lowest in various swab samples (35.4%). In contrast, no significant differences were found among sampling types in subclinically infected farms (*p* = 0.513), where serum samples showed the highest prevalence (13.7%), followed by swab samples (10.8%), with the lowest in tissue samples (8.3%).

The subgroup analysis based on diagnostic methods revealed substantial differences in *M. hyopneumoniae* prevalence (*p* = 0.000). The infection rate detected by antibody-based methods was 40.2%, while antigen-based methods yielded a lower prevalence of 20.2%. The highest prevalence was observed with histopathological examination, primarily based on pathological tissue samples, at 63.1%. Diagnostic heterogeneity should therefore be considered when interpreting prevalence estimates, and future studies would benefit from standardized, validated protocols. In addition, slaughter lesion scoring data from Chinese studies indicate that using the Combined Lung and Pleural (CLP) scoring method on 12,630 lungs across 247 batches reported median bronchopneumonia lesions in approximately 59.7% of lungs, with an average affected lung surface area of 3.66%, a bronchopneumonia lesion area of 5.8%, a scar tissue proportion of 19.8%, pleuritis on apical and cardiac lobes at 0.62%, diaphragmatic pleuritis at 4.07%, and an APPI of 0.12 [77].

### 3.5. Publication Bias and Sensitivity Analysis

**Figure 4:**
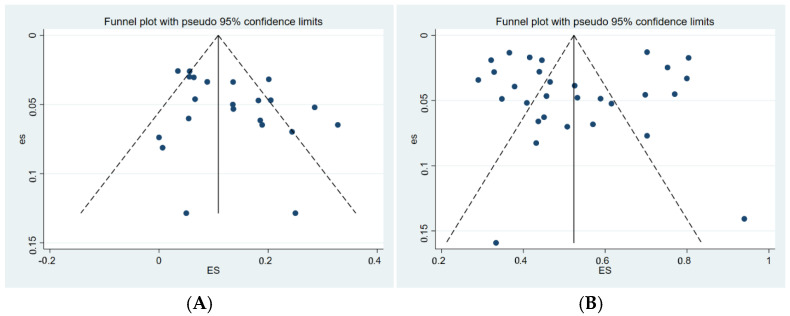
Funnel plot showing publication bias in studies reporting the prevalence of *M. hyopneumoniae* in subclinical infection swine farms (**A**) and clinical infection swine farms (**B**).

Funnel plots were used to assess potential publication bias among the included studies. Both the funnel plot for subclinically infected swine farms (Figure 4A) and that for clinically infected swine farms (Figure 4B) exhibited noticeable asymmetry (represented by dots), indicating a potential presence of publication bias in the included literature.

**Figure 5:**
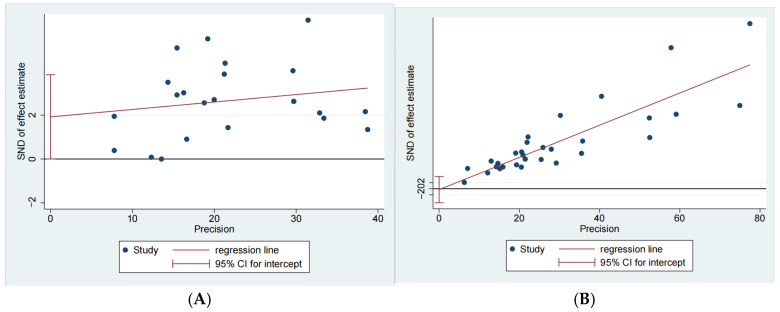
Egger test for publication bias in epidemiological studies of *M. hyopneumoniae* in subclinical infection swine farms (**A**) and clinical infection swine farms (**B**). SND: standard normal deviate; precision: inverse of the variance.

Egger’s test was further applied to evaluate publication bias among the included studies. The results indicated a significant publication bias (*p* = 0.050, Figure 5A; *p* = 0.000, Figure 5B) [78].

**Figure 6:**
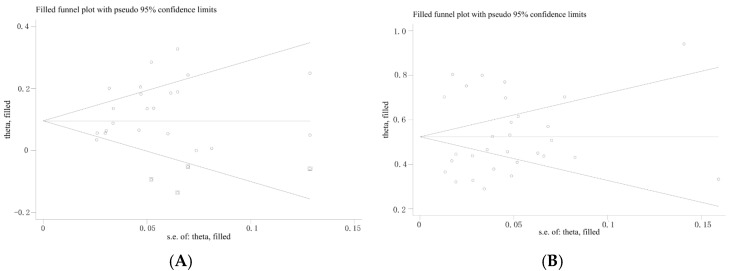
Filled funnel plots for subclinical infection swine farms (**A**) and clinical infection swine farms (**B**) with the Trim-and-Fill Method. ◯ indicates observed studies; ⌼ indicates missing studies.

Subsequently, the Trim-and-Fill method was employed to further assess the impact of publication bias on the results [21,22]. After adding six imputed studies for subclinically infected swine farms and none for clinically infected swine farms, the meta-analysis was re-conducted, yielding an adjusted *p*-value of 0.000 (Figure 6). The heterogeneity remained significant, indicating that the corrected results are robust.

**Figure 7:**
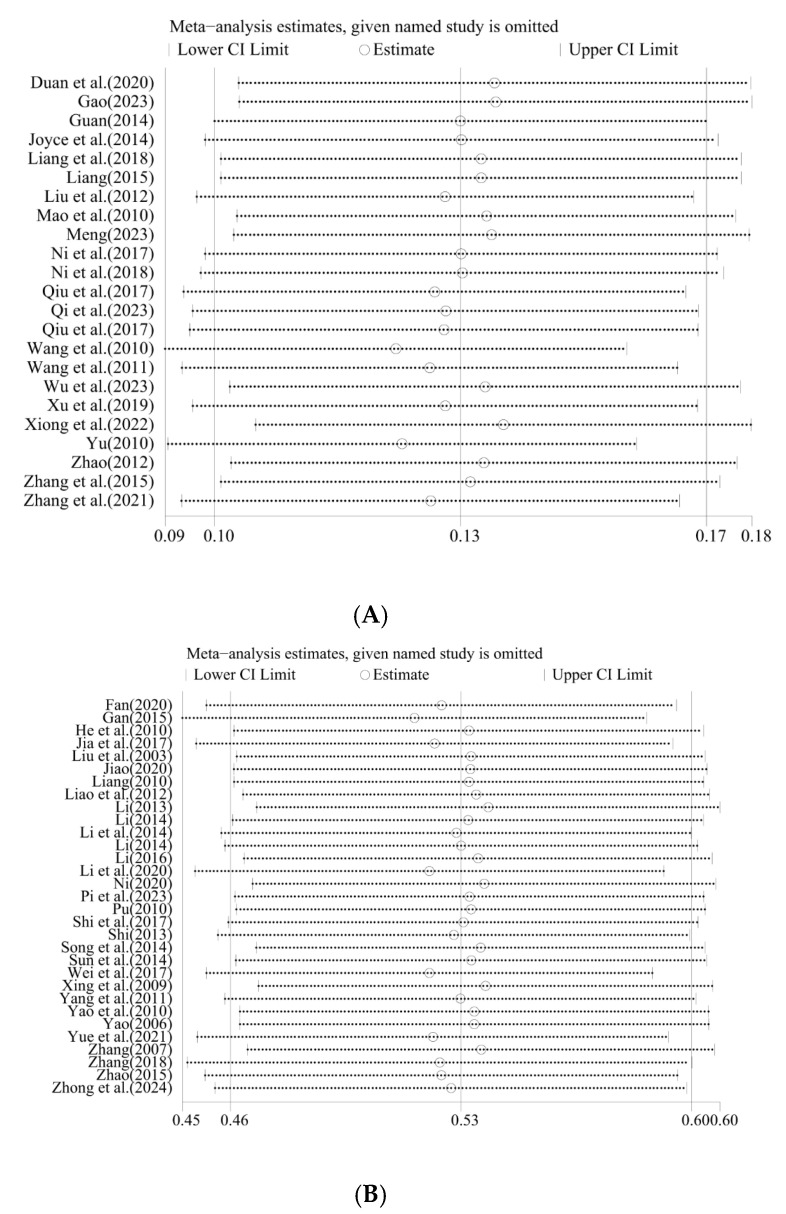
Leave-one-out sensitivity analysis of epidemiological studies on *M. hyopneumoniae* in subclinical infection swine farms (**A**) and clinical infection swine farms (**B**) (the circles indicate the pooled relative risk estimate when each individual trial is omitted).

In this study, the sensitivity analysis (leave-one-out sensitivity analysis) revealed that omitting any single study did not substantially affect the pooled effect size or its 95% confidence interval (Figure 7). This indicates that the meta-analysis results are not overly influenced by any individual study. The overall consistency of the estimates across all iterations supports the robustness and reliability of the findings.

## 4. Discussion

*M. hyopneumoniae* prevalence in domestic swine herds varies widely across countries that employ modern swine production systems, ranging from 30% to 80%, depending on the region and diagnostic methods used. In Spain and Portugal, 31% of slaughtered swine exhibited lung lesions compatible with the pathogen, and 24.2% tested positive by PCR [79]. In Argentina, molecular detection revealed a prevalence of 48% in fattening swine [80]. In Belgium and the Netherlands, infection rates among young swine ranged from 7.1% to 10.9% [81]. Recent surveillance in the United States has shown that although the infection rate in breeding herds decreased from 21.75% in 2021 to 13.5% in 2022 due to enhanced vaccination and biosecurity measures, a resurgence was observed in growing swine during the wean-to-finish phase in 2024 [82]. As of 2024, *M. hyopneumoniae* remains a major concern in China’s swine industry. A study analyzing 989 samples from 27 provinces between 2018 and 2020 reported an increasing positivity rate from 7.2% in 2018 to 18.4% in 2019, and 43.8% in 2020 [72]. Nevertheless, as the distribution of samples across provinces was uneven and the sample size in several provinces was relatively limited, these data should be interpreted with caution, since they may not fully reflect the true prevalence at the provincial level. Compared with these countries, mainland China shows a relatively higher overall burden of *M. hyopneumoniae*, particularly in small-scale farms and provinces with harsher climates such as the Northwest and Qinghai–Tibet Plateau. These international comparisons indicate that while the general epidemiological patterns of *M. hyopneumoniae* are broadly similar worldwide, the scale, persistence, and regional heterogeneity of infection in mainland China reflect unique challenges that require region-specific control strategies.

Subclinical infections, often asymptomatic, may evade detection while still contributing to disease transmission. In contrast, clinically affected farms exhibit overt respiratory symptoms, resulting in higher detection rates. Given the marked differences in clinical manifestations and epidemiological characteristics of *M. hyopneumoniae* infections across swine farms in mainland China, this study stratified farms into subclinical and clinical infection categories to better reflect infection dynamics and improve the accuracy of pooled prevalence estimates. This stratification not only enhances epidemiological insight but also informs the development of targeted control strategies. As highlighted by [83,84], classification based on infection status is essential for effective disease surveillance and intervention planning. By adopting this approach, our study provides a more nuanced understanding of *M. hyopneumoniae* prevalence in both subclinical and clinical settings.

To the best of our knowledge, this is the first systematic review and meta-analysis to evaluate the pooled prevalence of *M. hyopneumoniae* in mainland China. A comprehensive analysis of studies published between 2003 and 2024 was conducted, and 54 eligible articles were included. The aggregated data suggest that the estimated overall prevalence of *M. hyopneumoniae* infection in swine farms in mainland China is 33.4%. Stratified analysis indicates prevalence rates of 11.5% in subclinically infected farms and 52.9% in clinically infected farms.

The infection rates of *M. hyopneumoniae* in clinically infected swine farms were significantly higher than those in subclinically infected farms across all age groups. In clinical farms, the highest prevalence was observed in breeding and fattening swine (>49%), which was significantly greater than in the other three age groups. This suggests an age-related increase in *M. hyopneumoniae* infection, with breeding and fattening swine identified as high-risk groups. Previous studies have demonstrated a similar trend. A large-scale study conducted in Belgium and the Netherlands reported that *M. hyopneumoniae* prevalence increased from 8.5% in 3–5-week-old piglets to 16.2% in 6–11-week-old swine, and up to 53.4% in 12–25-week-old fattening swine [5], indicating a clear age-related rise in infection. In contrast, the prevalence across all age groups in subclinical farms remained below 21%. The high I^2^ values observed indicate substantial heterogeneity among studies, potentially attributable to differences in diagnostic methods, geographic regions, and sample sources. Therefore, these results should be interpreted with caution.

The infection rate of *M. hyopneumoniae* in clinically infected swine farms varies significantly across different regions and is markedly higher than that in subclinical farms. Among clinical infection farms, regional differences are significant, with the highest infection rates observed in the Northwest region, followed by the Qinghai–Tibet Plateau and northern regions, while southern regions exhibit relatively lower infection rates. However, in contrast to clinical farms, subclinical farms exhibited minimal regional variation, with rates fluctuating between 9% and 12% [72].

The higher prevalence of *M. hyopneumoniae* in the Northwest and Qinghai–Tibet Plateau regions may be attributed to the predominance of free-range and small-scale farming practices and relatively weak biosecurity measures, and scarce veterinary support facilitates transmission. By contrast, southern regions are dominated by large-scale commercial farms with stricter management and vaccination, resulting in lower prevalence. These findings underscore how the dual structure of pig farming in mainland China drives regional disparities and highlight the need for tailored control strategies that consider both production systems and local conditions. Climatic factors also play a crucial role in the epidemiology of *M. hyopneumoniae*. Harsh environmental conditions characterized by aridity, low temperatures, and large diurnal temperature variations can impair the immune function of swine and enhance the pathogenicity, thus increasing infection rates. Conversely, in some southern regions, despite limited management, higher humidity and warmer climates may influence pathogen survival and transmission differently, resulting in complex regional infection patterns [69]. In addition, provinces in southern China, where industrialized farming is more advanced and infrastructure is better developed, generally reported lower prevalence. Northern provinces, which combine both smallholder and intensive farms, showed intermediate values. Therefore, control strategies for *M. hyopneumoniae* should be tailored to specific geographical areas and inter-provincial disparities by integrating local farming practices, climatic conditions, and management levels to develop scientifically sound and effective comprehensive prevention and control measures. Future research should emphasize longitudinal multi-regional and multi-seasonal studies to more thoroughly elucidate key factors influencing *M. hyopneumoniae* infection rates, thereby providing a theoretical basis for precision control.

Subgroup analysis based on farm scale revealed significant differences in *M. hyopneumoniae* infection rates among swine farms in mainland China. The highest prevalence was observed in small-scale farms (35.7%), followed by medium-scale farms (27.9%), while large-scale farms had the lowest infection rate (2.3%). Large-scale intensive farms often implement strict biosecurity protocols—such as all-in/all-out systems, disinfection routines, and vaccination programs—reducing pathogen transmission [16]. They also benefit from better infrastructure and greater investment in disease prevention [85]. In contrast, smallholder farms typically lack proper facilities, have inadequate hygiene and low vaccination coverage, and maintain frequent contact with external animals, people, or equipment, all of which increase the risk of infection [86,87]. These conditions increase the risk of *M. hyopneumoniae* transmission not only within farms but also across regions, since biosecurity barriers are insufficient to prevent the circulation of respiratory pathogens. The limited implementation of all-in/all-out management and the mixing of animals of different origins further exacerbate the persistence of infection in small-scale systems. In mainland China, this dual structure presents unique epidemiological challenges, highlighting the need for differentiated strategies that account for farm scale and management level.

Inadequate disease monitoring further hampers timely control. However, a larger farm scale does not necessarily eliminate the risk of infection. The critical factor lies in implementing scientific husbandry practices and stringent biosecurity measures.

Subgroup analysis by sample type revealed significant differences among clinically infected swine farms (*p* = 0.033). The highest infection rate was detected in tissue samples (56.8%), followed by serum samples (44.6%), with the lowest in various swab samples (35.4%). In contrast, no significant differences were found among subclinically infected farms (*p* = 0.513), with serum, swabs, and tissue showing infection rates of 13.7%, 10.8%, and 8.3%, respectively. The reported prevalence of *M. hyopneumoniae* infection is significantly influenced by sample type. Common specimens include lung tissue, bronchoalveolar lavage fluid (BALF), nasal swabs, tonsillar swabs, and oral fluids. Lung tissue and BALF, which reflect infection at the primary colonization sites, often yield higher detection rates than upper respiratory tract samples due to higher bacterial load [86,88]. In contrast, nasal or tonsillar swabs may result in false negatives, particularly in subclinical or chronic infections [89]. Oral fluids are useful for herd-level surveillance but have limited sensitivity for detecting individual infections [90]. These differences in detection rates highlight the need for careful sample selection based on diagnostic goals. Studies show that relying solely on non-invasive sampling may underestimate true prevalence, affecting epidemiological assessments and control strategies [91,92]. Therefore, incorporating more sensitive sample types, such as BALF or postmortem lung tissue, is recommended for accurate prevalence estimation.

Subgroup analysis by diagnostic method revealed significant differences in the detection rates of *M. hyopneumoniae* among testing approaches. In this study, three categories of diagnostic methods were used to detect *M. hyopneumoniae*: antigen detection methods (PCR and AGPT), antibody detection methods (ELISA and agglutination tests), and histopathological examination. PCR and AGPT directly detect the pathogen and are highly sensitive during the acute infection phase but may yield false negatives due to low pathogen load or poor sampling technique [86]. In contrast, ELISA and agglutination tests detect antibodies and are useful for assessing previous exposure or vaccination response, although they are limited by the window period before seroconversion [93]. Histopathology, while valuable for confirming chronic or late-stage lesions, requires postmortem samples and may not differentiate *M. hyopneumoniae* from other respiratory pathogens without specific staining [94]. These discrepancies reflect differing stages of infection. Such patterns help interpret diagnostic results relative to infection stage: PCR-positive but ELISA-negative results indicate early infection, whereas ELISA-positive but PCR-negative results suggest past infection or vaccination [95]. Although CLP-based lesion scoring provides valuable confirmation of subclinical infections in Chinese pig herds, such data remain scarce. More systematic, abattoir-based surveillance is needed to complement laboratory-based prevalence estimates. In general, differences among sample features strongly influence prevalence estimates of *M. hyopneumoniae*. Tissue samples usually yield higher detection rates as they represent the primary site of infection, while serum mainly reflects past exposure, and swabs tend to underestimate prevalence due to low and variable bacterial load. Moreover, the diagnostic method also plays a role, with PCR and histopathology detecting active infection, whereas serology reflects prior infection or vaccination. Therefore, combining molecular, serological, and pathological approaches provides a more accurate diagnosis and comprehensive understanding of infection dynamics in a herd.

The outcomes of this meta-analysis carry several important implications. First, the identification of higher prevalence in clinically affected herds, small-scale farms, and specific regions highlights priority targets for intervention. Region-specific vaccination programs, enhanced biosecurity, and improved diagnostic strategies are essential to reduce transmission. Second, the persistence of infection in smallholder systems indicates that national policies should support these farms through education, subsidies for vaccination, and better access to veterinary services. Finally, the study provides an evidence-based foundation for policymakers and industry stakeholders to optimize resource allocation, improve herd health management, and mitigate the substantial economic losses caused by *M. hyopneumoniae* in mainland China.

There are several limitations to this study related to the quality, consistency, and geographic coverage of the included literature. Uncontrolled confounders (e.g., vaccination status, animal health, and sampling protocols) limit causal inferences. Limited studies from certain regions (e.g., Tibet, Ningxia, and Hainan) reduce geographic representativeness, and therefore the results should be interpreted with caution for these areas. High variability in diagnostic methods, including differences in sensitivity and specificity among PCR, ELISA, AGPT, and histopathology, together with the unclear distinction between vaccine- and infection-induced antibodies, may affect the accuracy and comparability of prevalence estimates.

## 5. Conclusions

This meta-analysis reveals a high and variable prevalence of *M. hyopneumoniae* in swine farms in mainland China, particularly in clinically infected herds, small-scale operations, and certain regions. These findings call for targeted control strategies considering farm type, swine age, climate, and diagnostic methods. Enhancing biosecurity, improving diagnostics, and tailoring regional vaccination and management programs are essential. Future research should prioritize longitudinal monitoring and integrated control efforts to address the complex nature of *M. hyopneumoniae* transmission.

## Figures and Tables

**Table 1:** Baseline data of the studies included in the meta-analysis.

No.	Authors	Publication Year	Diagnostic Technique	Sample Type	No. of Positive Samples	No. of Samples
1	Duan et al. [23]	2020	PCR	Pathological material	62	1114
2	Fan [24]	2020	AGPT, histopathological examination	Pathological material	118	168
3	Gan [25]	2015	PCR	Nasal swabs	47	50
4	Gao [26]	2023	PCR	Throat swabs	83	1478
5	Guan [27]	2014	PCR	Lung	0	183
6	He et al. [28]	2010	ELISA	Serum	210	460
7	Jia et al. [29]	2017	Agglutination test	Serum	1233	1639
8	Jiao [30]	2020	Histopathological examination, PCR	Lung, throat swabs	1119	1375
9	Joyce et al. [31]	2014	PCR	Nasal swabs	54	399
10	Li [32]	2016	PCR, ELISA	Lung, nasal swabs	245	647
11	Li [33]	2013	PCR, ELISA	Pathological material, serum, nasopharyngeal swabs	247	852
12	Li et al. [34]	2020	Histopathological examination	Lung	730	917
13	Li, Y.Y. [35]	2014	PCR	Lung	364	782
14	Li, S. [36]	2014	ELISA, PCR	Serum, lung	352	670
15	Li et al. [37]	2014	PCR	Lung	122	214
16	Liang [38]	2015	ELISA	Serum	78	882
17	Liang [39]	2010	ELISA	Serum	210	460
18	Liang et al. [40]	2018	ELISA	Serum	49	619
19	Liao et al. [41]	2012	ELISA	Serum	13	39
20	Liu et al. [42]	2012	PCR	Serum	15	60
21	Liu et al. [43]	2003	ELISA	Serum	63	146
22	Mao et al. [44]	2010	PCR	Pathological material	1	151
23	Meng [45]	2023	PCR	Serum	69	1080
24	Ni et al. [46]	2017	PCR	Nasal swabs	48	352
25	Ni et al. [47]	2018	PCR, ELISA	Nasal swabs, serum	119	876
26	Ni [48]	2020	PCR, ELISA	Nasal swabs, serum, lung, tracheal swab	414	1260
27	Pi et al. [49]	2023	PCR	Nasal swabs, lung	114	253
28	Pu [50]	2010	ELISA	Serum	100	229
29	Qi et al. [51]	2023	PCR	Lung, nasal swabs	49	264
30	Qiu et al. [52]	2017	ELISA	Serum	93	454
31	Qiu et al. [53]	2017	PCR	Nasal swabs, tracheal swab, alveolar lavage fluid	82	450
32	Shi [54]	2013	ELISA	Serum	249	423
33	Shi et al. [55]	2017	ELISA	Serum	103	203
34	Song et al. [56]	2014	ELISA	Serum	2056	5619
35	Sun et al. [57]	2014	ELISA	Serum	562	1280
36	Wang et al. [58]	2011	PCR	Lung	50	205
37	Wang et al. [59]	2010	PCR	Lung, nasal swabs, tracheal swab, alveolar lavage fluid	78	238
38	Wei et al. [60]	2017	Agglutination test	Serum	2687	3343
39	Wu et al. [61]	2023	PCR	Lung, nasal swabs	31	469
40	Xing et al. [62]	2009	AGPT	Pathological material	886	2757
41	Xiong et al. [63]	2022	PCR	Serum	52	1500
42	Xu et al. [64]	2019	PCR	Pathological material	45	238
43	Yang et al. [65]	2011	ELISA, PCR	Serum, lung	232	436
44	Yao [66]	2006	ELISA	Serum	152	372
45	Yao et al. [67]	2010	Histopathological examination, PCR, ELISA	Lung, serum, nasopharyngeal swabs	1451	3492
46	Yu [68]	2010	ELISA	Serum	105	368
47	Yue et al. [69]	2021	PCR	Lung	378	491
48	Zhang [70]	2018	Histopathological examination, PCR	Serum, lung	4214	6000
49	Zhang [71]	2007	ELISA	Serum	146	420
50	Zhang et al. [72]	2021	PCR	Pathological material	199	989
51	Zhang et al. [73]	2015	PCR	Pathological material	3	60
52	Zhao [74]	2012	PCR	Lung, nasal swabs	16	276
53	Zhao [75]	2015	Histopathological examination	Lung	335	480
54	Zhong et al. [76]	2024	ELISA	Serum	224	364

Antibody tests: ELISA and agglutination test; antigen tests: PCR and AGPT (agar gel precipitation test). Swabs: nasal, throat, nasopharyngeal, and tracheal. Lung: lung tissue samples.

**Table 2:** Results of subgroup analysis of data on the prevalence of *M. hyopneumoniae* infection.

Subgroup		No. of Studies	No. of Positive Samples	No. of Samples	Infection Rate		Heterogeneity			P
					Estimates	95% CI	X^2^	*p*-Value	I^2^/%	
Age					39.80%	[0.341; 0.456]				
Subclinical infection swine farms	Suckling swine	3	25	144	0.137	[0.000; 0.480]	/	/	/	0.606
	Nursery swine	2	19	80	0.207	[0.132; 0.305]	/	/	/	
	Growing swine	3	45	387	0.117	[0.052; 0.201]	/	/	/	
	Fattening swine	5	61	334	0.169	[0.080; 0.282]	16.272	0.001	81.563	
	Breeding swine	3	36	220	0.172	[0.085; 0.277]	6.943	0.074	56.790	
Clinical infection swine farms	Suckling swine	18	1878	3812	0.380	[0.246; 0.523]	1246.462	0.000	98.636	0.005
	Nursery swine	11	1585	3625	0.361	[0.161; 0.589]	1753.059	0.000	99.430	
	Growing swine	16	1146	3996	0.327	[0.235; 0.426]	570.102	0.000	97.369	
	Fattening swine	21	3459	7683	0.491	[0.364; 0.619]	2363.728	0.000	99.154	
	Breeding swine	18	2378	4719	0.617	[0.495; 0.734]	994.129	0.000	98.189	
Period					29.80%	[0.232; 0.367]				
Subclinical infection swine farms	Before 2013	7	240	1642	0.107	[0.031; 0.218]	223.640	0.000	97.317	0.944
	2014–2018	10	633	5712	0.110	[0.084; 0.139]	158.201	0.000	89.254	
	2019–2024	7	458	5376	0.124	[0.066; 0.197]	342.961	0.000	97.959	
Clinical infection swine farms	Before 2013	16	9982	20,737	0.497	[0.394; 0.600]	3046.162	0.000	99.508	0.803
	2014–2018	10	7830	12,911	0.546	[0.430; 0.659]	1329.997	0.000	99.323	
	2019–2024	6	1668	3560	0.562	[0.234; 0.862]	1946.302	0.000	99.743	
Region					33.40%	[0.256; 0.416]				
Subclinical infection swine farms	Northern region	2	93	942	0.094	[0.076; 0.114]	/	/	/	0.272
	South region	13	682	5716	0.096	[0.056; 0.145]	434.194	0.000	96.776	
	Qinghai–Tibet Plateau region	2	131	1069	0.118	[0.099; 0.138]	/	/	/	
Clinical infection swine farms	Northern region	6	4416	9447	0.557	[0.375; 0.731]	1465.31	0.000	99.659	0.005
	South region	13	2595	6144	0.474	[0.418; 0.530]	208.639	0.000	94.248	
	Qinghai–Tibet Plateau region	1	249	423	0.589	[0.541; 0.635]	/	/	/	
	Northwest region	7	5470	7468	0.642	[0.538; 0.739]	434.408	0.000	98.619	
Farming scale					25.60%	[0.160; 0.365]		0.000		
Small-scale swine farms		8	362	1974	0.357	[0.192; 0.541]	531.799	0.000	98.308	0.012
Medium-scale swine farms		3	243	1560	0.279	[0.062; 0.573]	297.666	0.000	98.992	
Large-scale swine farms		3	119	1076	0.023	[0.000; 0.169]	/	/	/	
Season					31.30%	[0.209; 0.428]				
Subclinical infection swine farms	Spring	3	170	902	0.166	[0.054; 0.321]	/	/	/	0.127
	Summer	2	33	720	0.044	[0.030; 0.061]	/	/	/	
	Autumn	2	29	570	0.050	[0.034; 0.070]	/	/	/	
	Winter	3	123	1024	0.094	[0.020; 0.215]	/	/	/	
Clinical infection swine farms	Spring	6	655	1719	0.547	[0.259; 0.819]	/	/	/	0.105
	Summer	4	775	1166	0.360	[0.107; 0.664]	765.055	0.000	99.346	
	Autumn	3	488	1215	0.651	[0.564; 0.733	23.081	0.000	87.002	
	Winter	3	221	527	0.471	[0.309; 0.636]	/	/	/	
Sampling type					31.60%	[0.260; 0.374]				
Subclinical infection swine farms	Serum	8	544	5486	0.137	[0.082; 0.203]	283.042	0.000	97.527	0.513
	Swab	7	295	2728	0.108	[0.069; 0.155]	77.43	0.000	90.96	
	Tissue	10	386	3210	0.083	[0.030; 0.156]	315.742	0.000	97.15	
Clinical infection swine farms	Serum	21	9109	21,068	0.446	[0.369; 0.525]	2415.530	0.000	99.172	0.033
	Swab	8	1570	4001	0.354	[0.252; 0.462]	246.880	0.000	96.760	
	Tissue	14	6257	11,199	0.568	[0.449; 0.683]	1791.307	0.000	99.274	
Diagnostic method					24.20%	[0.192; 0.296]				
Antibody testing		26	9619	20,973	0.402	[0.131; 0.494]	4408.821	0.000	99.410%	0.000
Antigen testing		33	5537	19,256	0.202	[0.137; 0.275]	8490.069	0.000	99.282%	
Histopathological testing		5	4637	7111	0.631	[0.463; 0.783]	811.349	0.000	99.384%	

## Data Availability

The original contributions presented in this study are included in the article/Supplementary Material. Further inquiries can be directed to the corresponding author(s).

## Supplementary Materials

The following supporting information can be downloaded at: https://www.mdpi.com/article/10.3390/vetsci12090863/s1.
